# Modifying Recycled Graphite with Co_3_O_4_ Towards a Novel, Efficient Anode for the Electrochemical Treatment of a Textile Dyebath

**DOI:** 10.3390/molecules31162785

**Published:** 2026-08-10

**Authors:** Milica Petrović, Slobodan Najdanović, Nena Velinov Georgiev, Jelena Mitrović, Miljana Radović Vučić, Miloš Kostić, Aleksandar Bojić

**Affiliations:** Department of Chemistry, Faculty of Sciences and Mathematics, University of Niš, 18000 Niš, Serbia; slobodan.najdanovic@pmf.edu.rs (S.N.); nena.velinov@pmf.edu.rs (N.V.G.); jelena.mitrovic1@pmf.edu.rs (J.M.); miljana.radovic@pmf.edu.rs (M.R.V.); milos.kostic@pmf.edu.rs (M.K.); aleksandar.bojic@pmf.edu.rs (A.B.)

**Keywords:** anode, graphite, modification, Co_3_O_4_, degradation, dye, water treatment

## Abstract

Electrochemical oxidation is an effective method for degrading environmentally harmful textile dyes that are difficult to remove by conventional treatments. The novel graphite–Co_3_O_4_ anode, prepared by electrochemically modifying recycled graphite tubes for AAS samples and characterized by SEM, EDX, FTIR, XRD, and BET, was used for electrochemical degradation of RB 4 dye in model solutions and textile dyeing effluent. Modification did not disrupt the graphite crystal structure but altered its surface properties, improving its dye degradation performance. It increased the decolorization rate constant of a model solution from 0.0148 min^−1^ to 0.0753 min^−1^ and maximum COD decay from 59% to 91%, reducing decolorization energy consumption from 3.26 kWh m^−3^ to 0.89 kWh m^−3^. Degradation at the graphite–Co_3_O_4_ anode proceeded via ·OH radicals and most likely the Co^3+^/Co^2+^ redox couple. It followed the pseudo-first-order kinetics. Pastel- and dark-shade dyeing textile effluents (containing 53 mg dm^−3^ and 159 mg dm^−3^ RB 4, respectively) were decolorized in about 70 and 110 min; COD decay reached about 87% and 80% after 180 min of electrolysis, respectively. The corresponding energy consumption was 1.75 kWh m^−3^, 2.39 kWh m^−3^, 2.97 kWh m^−3^, and 2.91 kWh m^−3^, respectively. The anode was efficient and stable in the given working conditions.

## 1. Introduction

The textile industry is one of the largest producers of industrial wastewater worldwide, characterized by a high proportion of synthetic dyes, various salts and additives, low transparency, high electrical conductivity, and a broad pH range, from acidic to alkaline. Its increasing production and water demand pose environmental concerns, and consequently, there is a need to treat this water before discharging it into natural water bodies [1,2].

The dyes used in the textile industry are a diverse group of synthetic organic compounds with various structures that exhibit high chemical stability under environmental conditions, during conventional water treatment, and in most aerobic biological processes [1,3]. Alternative treatments, such as various advanced oxidation processes (AOPs) and sorption, have been widely developed and improved over the years. However, despite their high efficiency in the dye removal process, their practical large-scale use is still challenging due to either their high cost and operational complexity (in the case of AOPs) or the disposal of the sludge (in the case of sorption) [1].

Electrochemical oxidation (EO) has shown strong potential for the removal of textile dyes. This technology is based on the generation of reactive oxygen or chlorine species at the anode by an electrical current, which attack the dye molecule and degrade it, completely or partially, due to their high oxidation potential. It has demonstrated many advantages, including high efficiency, relatively simple and low-cost equipment, and environmental friendliness. Its efficiency depends on multiple factors, the most important being the anode material [4]. It must be chemically inert and mechanically stable under conditions of high potentials and current densities and able to generate a high number of reactive species, such as hydroxyl radicals (OH) capable of degrading highly stable organics [1,2,3,4].

Based on the material and construction, several types of anodes have been developed for the electrooxidation of persistent organics, such as boron-doped diamond (BDD) [5,6,7], platinum [4,8], carbonaceous-based anodes, including graphite [4,9,10], lead oxide (PbO_2_) thin-film-based anodes [11,12], and dimensionally stable anodes (DSAs), based on metals such as RuO_2_, IrO_2_, SnO_2_, TiO_2_, Sb_2_O_3_, etc., and their mixtures [1,2,13,14,15]. Despite the generally high efficiency of these anodes, several challenges remain, including high costs, low mineralization, and mechanical instability in certain types of anodes. These issues, as well as the constant demand for higher efficiency and lower energy cost, led researchers toward new materials, i.e., modifications of existing ones, resulting in the development of various mixtures, composites, dopants, and other modifications for improving all types of anodes and thus the entire EO process [1,16,17,18,19].

Graphite electrodes exhibit high efficiency, low electrical resistivity, chemical stability, availability, low cost, and eco-friendliness. The possibility of improving their performance through modifications and preparation using recycled graphite from various sources makes graphite a versatile material with great potential for use as the anode in electrochemical processes, including EO [1,20,21].

Co_3_O_4_ and Co_3_O_4_-modified materials have demonstrated high catalytic potential in various AOPs, particularly in the photodegradation of organics, where they have actively participated in the degradation process. Co_3_O_4_ is a spinel-structured p-type semiconductor with a relatively narrow band gap and a balance of Co(II) and Co(III) ion contents that can be tailored using a synthetic procedure, which all make it relatively easy to excite by an external energy source. That, as well as the physicochemical stability, eco-friendliness, variety, simplicity, and low cost of preparation procedures [22,23,24,25,26], made it a choice for a graphite anode modification material to improve its efficiency and reduce energy consumption in the dye degradation process, which is the object of this work.

A Co_3_O_4_-modified graphite electrode was prepared by electrochemical deposition and used as the anode in the EO degradation of reactive dye. The objective of this work was to repurpose discarded graphite by modifying its surface to obtain a new, superior anode for the treatment of textile dye effluent. Discarded graphite tubes for AAS (atomic absorption spectroscopy) samples were used as the starting material for both non-modified and modified anodes. C.I. Reactive Blue 4 (RB 4) is an anthraquinone reactive dye containing a dichlorotriazine ring. It was chosen as a representative of an important group of textile dyes that are mostly used in dyeing cellulosic fibers [3,27]. The anode was characterized, and its efficiency was examined using RB 4. It was tested for the treatment of the textile dyebath (dyeing was carried out according to the real conditions recipe from [28]). The degradation mechanism, including the role of Co_3_O_4_, was proposed as a contribution to a deeper understanding of the mechanism(s) of these important types of EO processes. The kinetics model and effects of various operational parameters on dye removal and the chemical oxygen demand (COD) reduction efficiency, as well as energy consumption, were examined. Co_3_O_4_ modification improved the dye degradation and COD reduction efficiency and significantly reduced the energy consumption. The possibility of repurposing and reusing discarded graphite based on its activity improvement via a simple modification that brought a partial change to the mechanism of action, as well as its application in real conditions, represents the principal innovations of this research.

## 2. Results and Discussion

### 2.1. Characterization of the Anode

#### 2.1.1. Surface Morphology and Chemical Composition

**Figure 1:**
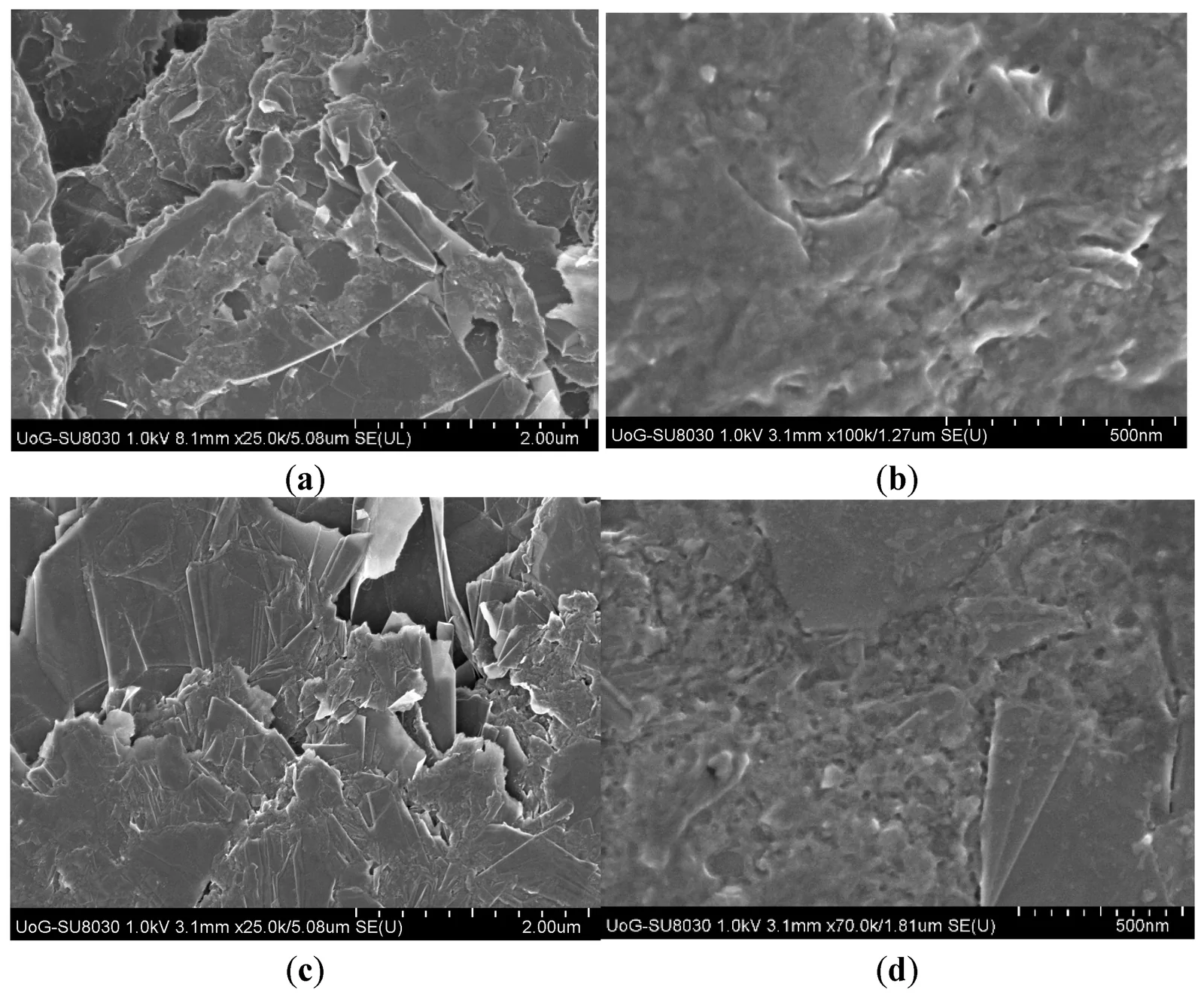
SEM images of (**a**,**b**) graphite anode and (**c**,**d**) graphite–Co_3_O_4_ anode at two different magnifications.

Scanning electron microscopy (SEM) images reveal a compact, predominantly two-dimensional structure of the graphite surface in both graphite and graphite–Co_3_O_4_ anodes (Figure 1a,d). Irregularly shaped layers are visible in several places, which might be a consequence of the electrochemical pretreatment of the anode’s surface, which caused the “peeling” of the graphitic surface in the form of foils. At higher magnification (Figure 1b,d), rare appearances of pit-like and hole-like structures smaller than 25 nm can be spotted in some places on the monolithic structure of both the graphite and graphite–Co_3_O_4_ anode surfaces. Though it is not a typical porous material, it does contain “holes” and “empty spaces” (which was analyzed by the BET method). The anodes have essentially the same surface morphology; the presence of Co_3_O_4_ did not cause any notable visual differences, indicating that it was incorporated in the graphite matrix at the molecular level rather than attached to the surface as a separate phase.

Energy-dispersive X-ray (EDX) analysis of the graphite anode has shown the presence of C and O (98.5 and 1.5 atomic%, respectively) on its surface. The presence of O was attributed to adsorbed water and other impurities. The graphite–Co_3_O_4_ anode’s surface contained C, Co, and O. The atomic% of Co varied between 11 and 16 on the examined surface, indicating that the surface was not fully covered with Co_3_O_4_, but it was relatively uniformly distributed. The O atomic% ranged from 18 to 29. Taking only the Co and O atoms, their atomic ratio would be between Co:O = 27.5:72:5 and 47:53. This deviates from their stoichiometric Co_3_O_4_ (Co:O = 57.14:42.86, Figure S1a). The variation and deviations were attributed to measurement uncertainty, surface contamination, such as adsorbed H_2_O or CO_2_, and possibly non-stoichiometry. Mapping of the present elements (Co, O, and C) on the examined surface is provided in the Supplementary Figure S1b, which shows a relatively uniform distribution of Co and O on the surface.

#### 2.1.2. Surface Functional Groups

**Figure 2:**
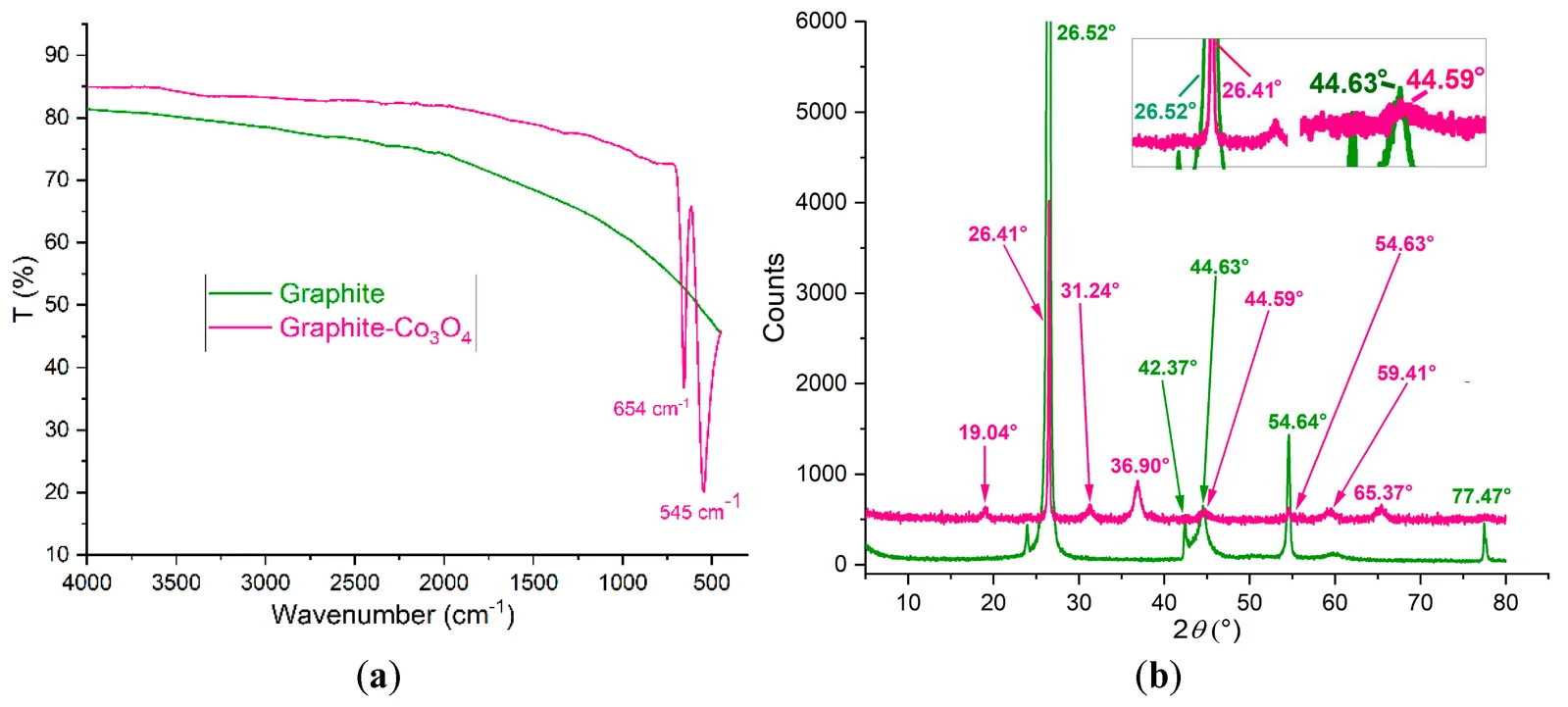
(**a**) FTIR spectra and (**b**) XRD pattern of graphite and graphite–Co_3_O_4_ anodes (two peaks in the XRD pattern are magnified to highlight the difference where possible).

There is a clear difference between the Fourier-transform infrared (FTIR) spectra of the graphite and graphite–Co_3_O_4_ anodes (Figure 2). The graphite anode surface practically did not show any absorption in the examined range (“FTIR silent”), indicating a highly pure and crystalline graphite surface without detected surface oxides. The visible bands are probably absent from the spectra because only vibrations that change the dipole moment are IR active (in pure graphite, all C atoms are sp^2^ hybridized, i.e., C-C bonds are highly symmetrical [29], and, in this case, it seems that potential surface impurities and oxides are not present in enough quantity to be detected by this technique). In the spectra of the graphite–Co_3_O_4_ anode, two strong narrow bands at 545 cm^−1^ and 654 cm^−1^ originating from the Co–O stretching vibrations of octahedral and tetrahedral cobalt ions, respectively, confirm the formation of spinel Co_3_O_4_ at the graphite surface [30,31].

#### 2.1.3. Crystal Structure of the Anodes’ Surface

The X-ray diffraction (XRD) pattern of the graphite anode contains sharp, narrow peaks at the following diffraction angles (2*θ* (°); corresponding crystal planes are given in brackets next to the 2*θ* value): 26.52° (002), 42.37° (100), 44.63° (101), 54.64° (004), and 77.47° (11) (110) (Figure 2a). They correspond to highly crystalline hexagonal 2H graphite (space group *P6_3_mc*, PDF 00-056-0159). The lattice parameters are *a* = 2.4607 Å and *c* = 6.7252 Å, with a crystallite size of 90.6 nm.

The XRD pattern of the graphite–Co_3_O_4_ anode contains the diffraction peaks originating from the graphite at 26.41° (002), 44.59° (101), and 54.63° (004) and the ones originating from the Co_3_O_4_ (space group *Fd3m*, PDF 01-074-1656) at 19.04° (111), 31.24° (320), 36.90° (311), 59.41° (511), and 65.37° (540) (Figure 2b). All the graphite peaks are notably weaker than those of pure graphite, and some of them disappeared from the pattern (or were at the noise level). This is probably due to the reduced weight ratio of graphite (dilution) because of the presence of the other phase, but other factors, such as surface coverage by the Co_3_O_4_ layer or changes in crystallinity, could also affect this phenomenon. The lattice parameters of the graphite phase are *a* = 2.5393 Å and *c* = 6.7196 Å, with a crystallite size of 62.8 nm, indicating somewhat disrupted crystallinity of the graphite surface. The peaks of the Co_3_O_4_ are broader than those of the graphite, indicating lower crystallinity of the Co_3_O_4_. The lattice parameter of the Co_3_O_4_ is *a* = 8.0789 Å, and the corresponding Co_3_O_4_ crystallite size is 11.4 nm. The graphite matrix was preserved after modification, but the graphite peaks were slightly shifted from their original positions, mostly towards lower diffraction angles, indicating a higher d-spacing. This, as well as the slight changes in the lattice parameters and the decreased crystallite size of the graphite, was attributed to the effect of the formation of Co_3_O_4_ in the graphite hexagonal matrix [32,33,34,35,36].

#### 2.1.4. Specific Surface Area Characteristics of the Anodes’ Surface

The Brunauer–Emmett–Teller (BET) surface area, as well as the pore volumes and pore sizes of the two anodes’ surfaces, are given in Table 1. Modification of the graphite surface with Co_3_O_4_ increased the specific surface area and pore volume by more than threefold, indicating an overall increase in surface porosity, which generally favors surface reactions. Based on the pore size, the surfaces of both anodes exhibit mesoporosity.

#### 2.1.5. Electrochemical Characterization of the Anodes

**Figure 3:**
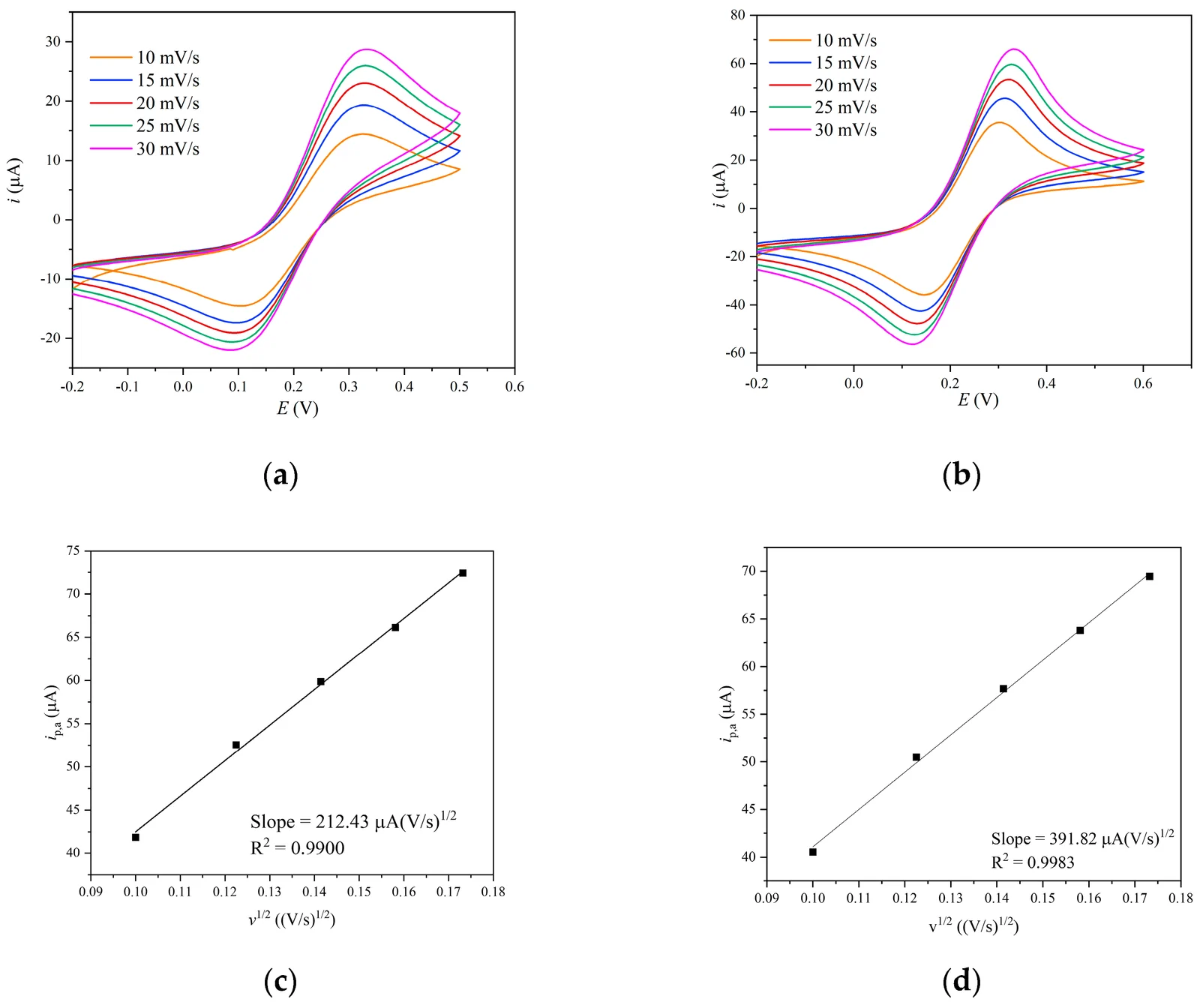
Cyclic voltammograms of (**a**) graphite and (**b**) graphite–Co_3_O_4_ anode at different scan rates and (**c**,**d**) their corresponding *i*_p,a_-versus-*v*^1/2^ dependence.

The cyclic voltammograms recorded in Fe(CN)_6_^3−/4−^ solution at different scan rates showed a progressive increase in both anodic and cathodic peak currents with an increasing scan rate for both graphite and graphite–Co_3_O_4_ anodes (Figure 3a,b). The well-defined oxidation and reduction peaks indicate that both anodes support electron transfer involving the ferri/ferrocyanide redox couple. The peak current notably increased at the Co_3_O_4_-modified anode. Using the slope obtained from the *i*_p,a_-versus-v^1/2^ dependence, the ESA of the graphite and graphite–Co_3_O_4_ anodes was calculated to be 0.0573 cm^2^ and 0.111 cm^2^, respectively, indicating that Co_3_O_4_ modification increased the ESA and improved the utilization of the anode interface (Figure 3c,d). This further indicates possible important involvement of the Co ions in the dye’s degradation.

### 2.2. Dye Degradation

#### 2.2.1. Comparison of the Non-Modified and Modified Anodes’ Performance

##### Decolorization

**Figure 4:**
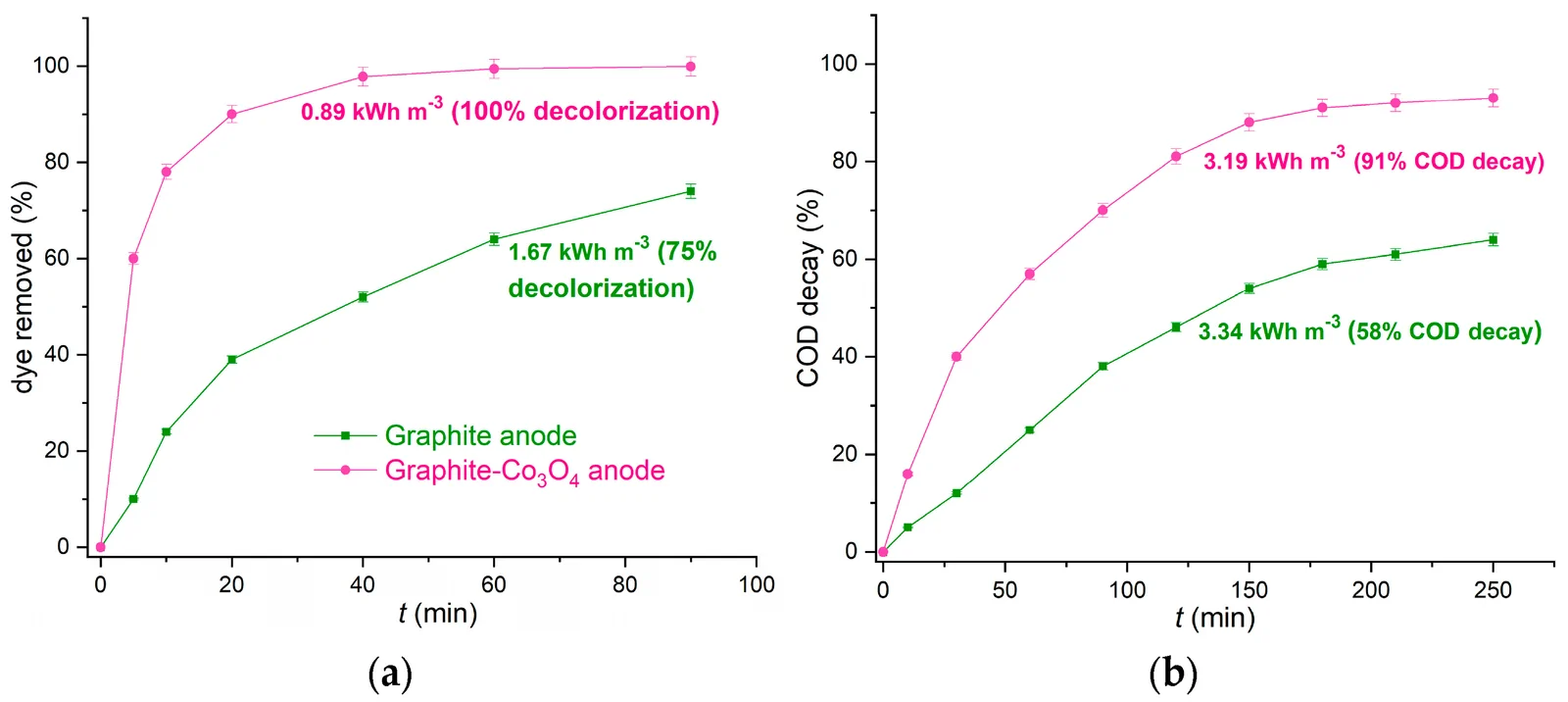
Comparison of (**a**) decolorization and (**b**) COD decay using graphite and graphite–Co_3_O_4_ anodes for degradation of RB 4 dye (*C*_0_ (RB 4) = 50 mg dm^−3^, *j* = 40 mA cm^−2^, *c* (Na_2_SO_4_) = 0.5 mol dm^−3^, and pH = 6.8). Energy consumption for the complete decolorization (**a**) and equilibrium COD decay after 180 min (**b**) of electrolysis at the two anodes is given next to the corresponding curves (all experiments performed in triplicate; estimated error within ±2%).

Figure 4a shows that Co_3_O_4_ modification significantly improved decolorization efficiency. After 20 min of electrolysis with the graphite–Co_3_O_4_ anode, 90% of the dye was removed, whereas only 39% was removed with the graphite anode for the same amount of time. After approximately 40 min of electrolysis, the dye was completely removed using the graphite–Co_3_O_4_ anode, whereas the removal efficiency with the graphite anode was about 55% over the same period. The corresponding rate constants for dye removal using graphite and graphite–Co_3_O_4_ anodes were 0.0148 min^−1^ and 0.0753 min^−1^, respectively, making the electrolysis with the modified anode more than 5 times faster.

##### Chemical Oxygen Demand (COD)

COD decay during degradation of the organic compound is attributed to the organic matter decay, i.e., its mineralization, and is an important indicator of that compound’s complete removal. During the first 60 min of electrolysis, COD decay of 25% and 57% was observed for the graphite and graphite–Co_3_O_4_ anodes, respectively. After 180 min of electrolysis, the COD decay for both anodes was approximately at its equilibrium, which was 59% and 91% for the graphite and graphite–Co_3_O_4_ anodes, respectively; further electrolysis did not bring significant improvement, being 64% and 93% after 250 min for the graphite and graphite–Co_3_O_4_ anodes, respectively (Figure 4b). The graphite–Co_3_O_4_ anode exhibited notably superior COD removal ability, meaning that modifying the graphite anode with Co_3_O_4_ significantly improved its degradation performance not only in terms of the degradation rate but also in terms of its overall degradation efficiency.

##### Energy Consumption

Energy consumption (*Ec*) was significantly lower when using the graphite–Co_3_O_4_ anode for both electrochemical decolorization and COD removal processes (Figure 4). The complete decolorization of RB 4 at the graphite anode took about 170 min. It required 3.26 kWh m^−3^, which was almost 3.7 times that for the complete decolorization with the graphite–Co_3_O_4_ anode under the same conditions (75% decolorization in this case happened relatively fast at the graphite anode (in about 60 min), and after that, it notably slowed down, and it was completed in about 170 min. That was why complete decolorization of this dye at this anode took so much energy compared to the 75% decolorization). *Ec* was also slightly lower for the 91% COD removal than for the 58% COD removal with the graphite and graphite–Co_3_O_4_ anodes, respectively (at 180 min of electrolysis, which was approximately the equilibrium time for COD removal). Modification of graphite with Co_3_O_4_ significantly reduced the *Ec* of anodic decolorization and COD reduction, mostly by reducing the time required for a certain process, i.e., improving the process’s efficiency. Reducing energy consumption is one of the most important achievements concerning the anode modification.

The *Ec* of RB 4 oxidative degradation at the graphite–Co_3_O_4_ anode and the *Ec* values of other relevant similar electrochemical degradation processes using different anodes are given in Table 2. Note that this is not an exact comparison of the efficiencies of different anodes but rather an approximate illustration of where the efficiency and possibilities of the graphite–Co_3_O_4_ anode currently stand.

#### 2.2.2. Radical Scavenger Tests

Typical radical scavengers were used to determine which active oxygen species (typically generated during electrolysis in such systems) participated in dye degradation at both graphite and graphite–Co_3_O_4_ anodes and to what extent. For a graphite anode, the presence of isopropanol (IPA, ·OH scavenger) and 4,5-dihydroxy-1,3-benzendisulfonic acid (Tiron, superoxide ($O_{2}^{\cdot -}$) radical scavenger) reduced the reaction rate by 76% and 11%, respectively.

For a graphite–Co_3_O_4_ anode, the presence of isopropanol and 4,5-dihydroxy-1,3-benzendisulfonic acid reduced the reaction rate by 59% and 20%, respectively.

The presence of NaN_3_ (a singlet oxygen (^1^O_2_) scavenger) and catalase (an H_2_O_2_ scavenger) did not affect the reaction rate in either anode case. The results of radical scavenger tests are given in the Supplementary Figure S2, expressed as the ratio of the residual dye concentration at time *t* and the initial dye concentration (only the results for the radical scavengers that cause a decrease in the decolorization rate are presented to avoid redundant curves due to repetitiveness of the results).

Before these tests, electrochemical dye degradation was performed in an Ar-saturated atmosphere under the same conditions as in other experiments to check whether there was direct anodic oxidation, without the participation of dissolved oxygen. About 5% and 32% of the dye was degraded in 60 min of electrolysis at the graphite anode and the graphite–Co_3_O_4_ anode, respectively (Figure S3).

Electrolysis under an Ar-saturated atmosphere indicated that dye degradation via direct oxidation, i.e., via direct electron transfer from the dye molecule to the anode, was negligible in the case of the graphite anode, but it was notable using the graphite–Co_3_O_4_ anode.

Radical scavenger tests indicated that the singlet oxygen and H_2_O_2_ did not participate in dye degradation using either graphite or a graphite–Co_3_O_4_ anode. A superoxide radical had a slight effect on the degradation rate using a graphite anode and a very low effect on the degradation using a graphite–Co_3_O_4_ anode. Hydroxyl radical manifested by far the highest impact in both the graphite and the graphite–Co_3_O_4_ anode cases (a corresponding scavenger caused the most significant decrease in the degradation rate in both cases). It was a major dye degradation reagent in the case of the graphite anode; thus, it was assumed that dye degradation with the graphite anode proceeded via oxidation of the dye by anodically generated ·OH radicals by water oxidation at high potential [1]. It can be represented as follows:

(1) $$Graphite\overset{H_{2}O,\;\;\;high\;potential}{\to }Graphite\left(OH\right)$$

(2) $$Graphite\left(OH\right)\overset{Dye,\;\;\;high\;potential}{\to }\;Degradation\;products$$

Minor oxidation proceeded via superoxide radicals, according to the corresponding radical scavenger test:

(3) $$Graphite\overset{H_{2}O,\;\;\;high\;potential}{\to }Graphite\left(O_{2}^{\cdot -}\right)$$

(4) $$\;Graphite\left(O_{2}^{\cdot -}\right)\overset{Dye,\;\;\;high\;potential}{\to }\;Degradation\;products$$

Based on the radical scavenger test, the hydroxyl radical significantly affected dye degradation at the graphite–Co_3_O_4_ anode. However, that impact was notably lower than with the graphite anode, though the graphite–Co_3_O_4_ anode was more efficient. This indicates that Co_3_O_4_ significantly affected the dye degradation process, i.e., the redox couple Co(III)/Co(II) participated in the degradation via electron exchange as the mediator. This was also indicated by electrolysis in an Ar-saturated atmosphere, suggesting that direct oxidation of the dye by Co(III) ions could happen to some extent (knowing that they make the major difference between the two tested anodes), as well as by the electrochemical characterization of the anodes (which showed a higher electrochemical activity of the graphite–Co_3_O_4_ anode). The proposed probable oxidation mechanism could proceed via direct electron transfer from the dye to Co(III) and then from the as-generated Co(II) to the anode. That way, the Co(III) ions would be constantly replenished during the electrolysis process, which, in turn, would be facilitated by the presence of cobalt ions. The effect of superoxide in the dye oxidation at the graphite–Co_3_O_4_ anode is notably higher than at the graphite anode, indicating that the redox couple Co(III)/Co(II) emphasized the generation of superoxide radicals, which further oxidized the dye [23,38,39]. The three possible mechanisms of dye oxidation at the graphite–Co_3_O_4_ anode can thus be presented as follows:

(5) $$Graphite–{Co}_{3}O_{4}\overset{H_{2}O,\;\;\;high\;potential}{\to }Graphite–{Co}_{3}O_{4}\left(\cdot OH\right)$$

(6) $$Graphite–{Co}_{3}O_{4}\left(OH\right)\overset{Dye,\;\;\;high\;potential}{\to }Degradation\;products$$

(7) $$Graphite–{Co}_{3}O_{4}\to Graphite–{Co}_{3}O_{4}\left(dye\right)$$

(8) $$Graphite–{Co}_{3}O_{4}\left(dye\right)\overset{{Co}^{3+},\;\;\;High\;potential,}{\to }Degradation\;products$$

(9) $$Graphite–{Co}_{3}O_{4}\overset{{{Co}^{3+}H}_{2}O,\;\;\;high\;potential}{\to }Graphite–{Co}_{3}O_{4}\left(O_{2}^{\cdot -}\right)$$

(10) $$Graphite–{Co}_{3}O_{4}\left(O_{2}^{\cdot -}\right)\overset{Dye,\;\;\;high\;potential}{\to }Degradation\;products$$

Based on data from anodes’ characterization and radical scavenger tests, it was assumed that Co_3_O_4_ was formed on the graphite surface via cathodic electrodeposition and thermal treatment in air. Cobalt ions were attracted to the cathode by the negative potential, where they formed Co(OH)_2_ with OH^−^ ions generated at the cathode by the reduction of water. The following equations could represent the process [30,40]:

2H_2_O + 2e^−^ → H_2_ + 2OH^−^ (11)

Co^2+^ + 2OH^−^ → Co(OH)_2_ (12)

6Co(OH)_2_ + O_2_ → 2Co_3_O_4_ + 6H_2_O (13)

Co_3_O_4_ preserved its integrity, as did the graphite, but the overall modification altered the graphite surface, improving its electrochemical activity, with Co_3_O_4_ participating as the electrocatalyst in the dye degradation process. A significant increase in the specific surface area of the modified anode surface probably improved the dye sorption and thus enhanced the entire degradation process.

#### 2.2.3. Effect of the Initial Dye Concentration and Degradation Kinetics

Among the kinetic models tested, the pseudo-first-order model best described the electrochemical degradation of RB 4 at the graphite–Co_3_O_4_ anode, as indicated by the *R*^2^ values (Table 3).

The reaction rate constant decreased slightly with an increase in the initial dye concentration for the lower initial concentrations (about 8% for the increase from 20 mg dm^−3^ to 50 mg dm^−3^, Table 3) and more significantly for the higher initial concentrations within the range tested (for about 17% from 50 mg dm^−3^ to 100 mg dm^−3^ and 27% from 100 mg dm^−3^ to 200 mg dm^−3^). *R*^2^ values exceeded 0.97 across all initial concentrations examined, decreasing gradually with increasing initial concentration.

**Figure 5:**
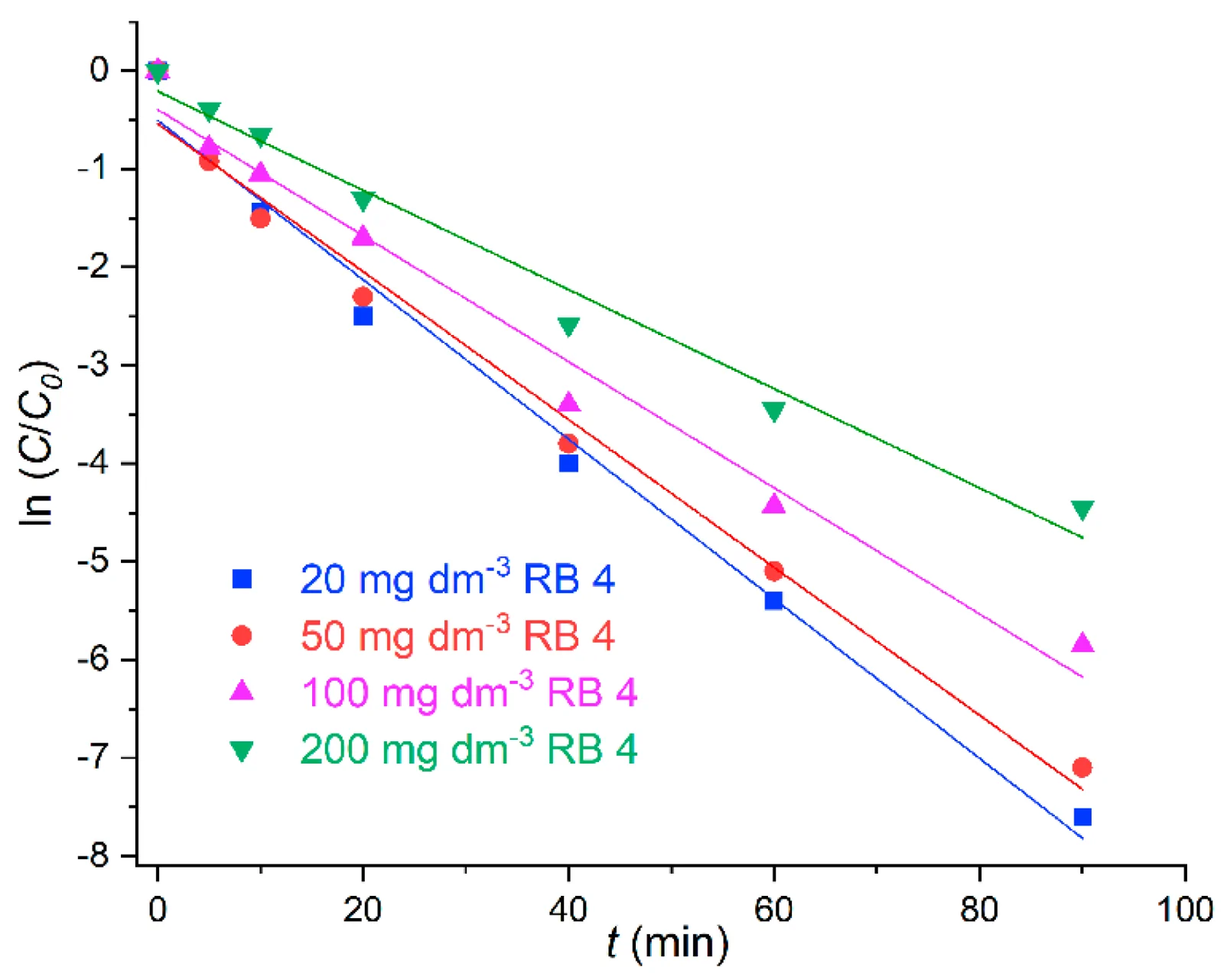
Pseudo-first-order kinetics of the electrochemical degradation of RB 4 at the graphite–Co_3_O_4_ anode.

Pseudo-first-order kinetics imply that the reaction proceeds according to the formula:

ln (*C/C*_0_) = −*k*_1_*t* (14)

where *C*_0_, *C*, and *k*_1_ are the initial dye concentration, the dye concentration at the reaction time *t* (mg·dm^−3^), and the reaction constant rate (min^−1^), respectively (Figure 5).

The pseudo-first-order kinetic model assumes that the concentration of one reagent (dye in this case) is so much lower than the concentration of the other (reactive oxidative species) that it can be considered constant. High *R*^2^ values indicate that the examined degradation reactions are well fitted by the model. The pseudo-first-order assumption is that the reaction rate constant should be independent of the initial dye concentration. However, it was not the case. It differed slightly in the lower concentration range and more significantly as the initial dye concentration increased. It implied that at some point, the initial dye concentration became significant and that the ratio “reactive oxidative species: dye” became low enough that it could not be considered constant anymore. That made it less favorable, thereby reducing the degradation rate constants. It also reflected the *R*^2^ values, which exceeded 0.98 for the lower initial concentration but fell below 0.98 for the higher initial concentration. It also indicated a certain deviation from pseudo-first-order kinetics, particularly for the higher concentrations tested. However, the *R*_2_ values remained high across the entire concentration range tested, so the degradation can be described by pseudo-first-order kinetics.

#### 2.2.4. Effects of Operational Parameters on the Degradation Rate at the Graphite–Co_3_O_4_ Anode

##### Effect of Current Density

**Figure 6:**
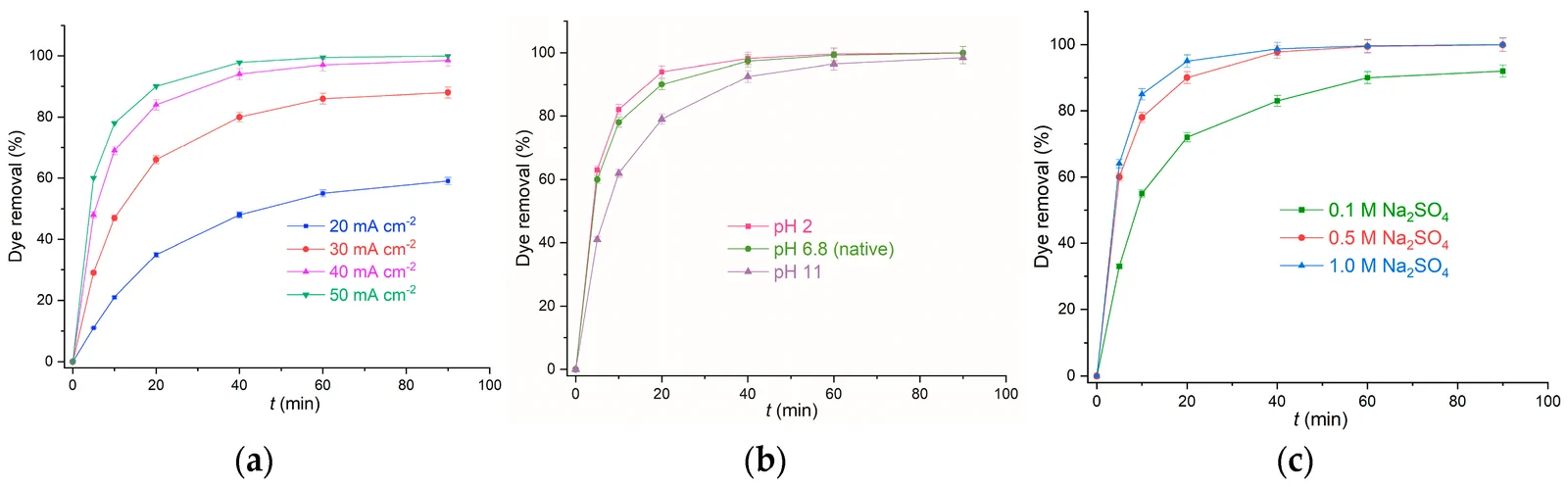
Effect of (**a**) current density, (**b**) pH, and (**c**) background electrolyte concentration on the decolorization of 50 mg dm^−3^ RB 4 solution (all experiments performed in triplicate; estimated error within ±2%).

The dye degradation rate and efficiency at the graphite–Co_3_O_4_ anode increased with increasing current density (Figure 6a). The increase was more pronounced at lower current densities. It became less significant as the current density increased from 30 mA cm^−2^ to 40 mA cm^−2^ and almost negligible when it increased from 40 mA cm^−2^ to 50 mA cm^−2^, especially at the longer electrolysis times. During the first 10 min, the degradation efficiency was 21%, 47%, 69%, and 77% at 20 mA cm^−2^, 30 mA cm^−2^, 40 mA cm^−2^, and 50 mA cm^−2^, respectively. Complete degradation was achieved at 40 mA cm^−2^ and 50 mA cm^−2^ (within the examined time range), and it was slightly faster at 50 mA cm^−2^: the corresponding rate constants for dye degradation at 40 mA cm^−2^ and 50 mA cm^−2^ were 0.0753 min^−1^ and 0.0782 min^−1^, respectively.

The dye degradation rate and efficiency strongly depend on the current density. It is a driving force in electrochemical reactions, which directly affects the production of oxidative species. Higher current densities mean higher anodic potentials and, accordingly, more intense production of oxidative species that can degrade a dye. In this case, it also means a higher probability of enhancing electron transfer from dye to cobalt ions. This directly reflected the dye degradation rate and efficiency, which increased significantly in the lower current density range but more slowly as the current density increased further. At some point, the degradation rate almost stopped increasing with further increases in the current density. It most likely happened because at higher current densities, side reactions, such as oxygen evolution and the generation of less active oxidative species (with lower ability to degrade a dye), became more intense [12,14]. Indeed, starting from 50 mA cm^−2^, the appearance of bubbles at the anode’s surface became notable. For these reasons, a current density of 40 mA cm^−2^ was selected for further experimentation.

##### Effect of pH

The decolorization rate at the graphite–Co_3_O_4_ anode decreased with increasing pH (Figure 6b). The decrease was only slight when changing the medium pH from acidic to neutral, and it became more significant in the alkaline medium. During about the first 30 min of electrolysis, decolorization proceeded a little faster in the acidic medium than in the neutral medium (for example, 94% and 90% of the color was removed at pHs 2 and 6.8 (solution’s native pH) in the first 20 min of electrolysis, respectively), but at about the 40th minute, the degradation rates had approximately equalized. At pH 11, 79% of the color was removed in the first 20 min of electrolysis, which was notably lower than at pHs 2 and 6.8. After 40 min of electrolysis, about 7.5% of the color remained at pH 11, while it was practically completely removed at lower pHs. The complete color removal at pH 11 was achieved after about 70 min of electrolysis.

The research covered a wide range of pH values, as these are found in real textile and other dye-containing effluents. The reaction rate was highest in an acidic medium, being just slightly faster than in a neutral medium, especially at shorter electrolysis times. The degradation efficiency was nearly identical, with the color removed in about 40 min of electrolysis in both acidic and neutral media. In an alkaline medium, the electrolysis was notably slower. Although the color removal efficiency was practically equal to that in the acidic and neutral media, complete color removal was achieved at a notably longer electrolysis time. So, in general, electrolytic dye degradation proceeded faster in the acidic–neutral medium (in which the pH did not have much of an impact) than in the alkaline one.

Several factors play a role in this case. In general, reactions at the interface are affected by the charges of the reagents. RB 4 dye has four p*Ka* values: 0.80, 1.44, 7.83, and 12.05 [27]. The pH_pzc_ of the graphite–Co_3_O_4_ anode’s surface was 8, meaning that it was positively charged below pH 8 and negatively charged above pH 8. According to the p*Ka* values of RB 4, it has four H^+^ dissociation sites, all negatively charged above pH 12. However, as the pH decreased, certain dissociation sites were protonated, depending on the ambient pH and specific p*Ka* values, so between pH 2 and 6.8 (tested in this work), three dye dissociation sites should be deprotonated, i.e., negatively charged. This emphasized the dye sorption at the positively charged anode’s surface via electrostatic interactions and, thus, the entire degradation reaction. The effect was more pronounced at a lower pH due to the expectedly more dominant positive charge at the anode’s surface, i.e., less pronounced as the pH was closer to 8. It seems that the medium H^+^ concentration did not play an important role in the active oxidative species in this case (acidic–neutral), which is consistent with some previous work [37,41]. However, other reports show a notably higher degradation rate and efficiency at pH 5 than at pH 3 [2] or at pH 2 than at pH 6 [14], indicating that the effect of pH on dye electrooxidation in acidic–neutral medium is not unambiguous and that it depends on multiple factors, which might be the dye’s molecular structure, the anode’s nature, degradation species, and others [2]. At pH 11, the anode’s surface is negatively charged, as is the dye molecule, which makes dye sorption more difficult. That might slow down the reaction. But there is a more pronounced aggravating factor affecting degradation under alkaline conditions: the ·OH radical can be scavenged by OH^−^ ions, leading to the formation of a weaker O-radical [42]. As shown, the OH radical is the major degrading reagent in this case; thus, depletion of the OH radicals slowed down dye degradation in an alkaline medium. In addition, O^−^ ions could adsorb on the Co_3_O_4_ surface and form a surface with the Co ions, making the surface processes less effective [39]. Nevertheless, the degradation process is effective in alkaline conditions, achieving complete decolorization (though not as much as in acidic–neutral conditions), which makes the graphite–Co_3_O_4_ anode suitable for dye degradation in a wide pH range, which can be found in real conditions.

##### Effect of Background Electrolyte Concentration

Na_2_SO_4_ was chosen as the background electrolyte because it is a commonly used salt in many textile dyeing processes involving reactive dyes [1,3]. Its initial concentration in the electrolysis solutions affected the decolorization process of RB 4 at the graphite–Co_3_O_4_ anode (Figure 6c). The decolorization rate and efficiency increased significantly by increasing the Na_2_SO_4_ concentration from 0.1 mol dm^−3^ to 0.5 mol dm^−3^. Further increases from 0.5 mol dm^−3^ to 1mol dm^−3^ improved the decolorization rate just slightly, while the decolorization efficiency remained unchanged (complete decolorization in about 40 min). During the first 20 min of electrolysis, 72%, 90%, and 95% of the color was removed with 0.1 mol dm^−3^, 0.5 mol dm^−3^, and 1 mol dm^−3^ Na_2_SO_4_, respectively. After 40 min of electrolysis, the color was completely removed at 0.5 mol dm^−3^ and 1 mol dm^−3^ Na_2_SO_4_. At the same time, 83% of the color was removed with 0.1 mol dm^−3^ Na_2_SO_4_, and the total removal efficiency with this concentration was about 92%.

The presence of electrolytes increased the solution’s electrical conductivity, i.e., reduced its resistance, thereby facilitating processes at the electrodes, in this case, the anodic generation of active oxidizing species that participated in dye degradation. It resulted in a notably higher decolorization rate and efficiency with 0.5 mol dm^−3^ Na_2_SO_4_ than with 0.1 mol dm^−3^ Na_2_SO_4_. In this range of electrolyte concentration, degradation increased with increasing background electrolyte concentration because it facilitated the production of degrading species at the anode at the same current density. Increasing the Na_2_SO_4_ concentration from 0.5 mol dm^−3^ to 1 mol dm^−3^ only slightly increased the degradation rate at shorter electrolysis times, and it did not improve the decolorization efficiency (complete decolorization was achieved at about the same time with both 0.5 mol dm^−3^ Na_2_SO_4_ and 1 mol dm^−3^ Na_2_SO_4_). So, at some point, the background electrolyte concentration stopped playing a significant role in emphasizing the decolorization process. Moreover, sulfate can also be oxidized at the anode, forming persulfate, according to the following equation [14]:

2SO_4_^2−^ → S_2_O_8_^2−^ + 2e^−^ (15)

This is a competitive reaction to the anodic production of the ·OH radicals, which, as shown, were the main degrading reagent. Generated persulfate is a much weaker oxidant than ·OH radicals; when the anodic generation of persulfate became considerably high, it affected the ·OH production and thus decolorization, which was not promoted by a further increase in Na_2_SO_4_ concentration.

Overall, the increase in Na_2_SO_4_ concentration increased electrical conductivity and promoted ·OH generation, thereby promoting decolorization. However, at some point, its side reactions became more prominent, reflecting the ·OH generation and decolorization process, which was not intensified anymore by further increases in Na_2_SO_4_ content.

#### 2.2.5. Treatment of the Textile Dyebath

The remaining dye concentrations in the textile effluents after finishing the dyeing procedure, removing the dyed fabric, cooling to 24 ± 1 °C, and electrolysis with the graphite–Co_3_O_4_ anode were 53 mg dm^−3^ (COD = 68.9 mg O_2_ dm^−3^) and 159 mg dm^−3^ (COD = 188.5 mg O_2_ dm^−3^) for pastel- and dark-shade dyeing effluents, respectively. The corresponding effluent pH was 11.54 and 11.29, and the electrical conductivity was 198 mS cm^−1^ and 201 mS cm^−1^ for pastel- and dark-shade dyeing effluents, respectively.

**Figure 7:**
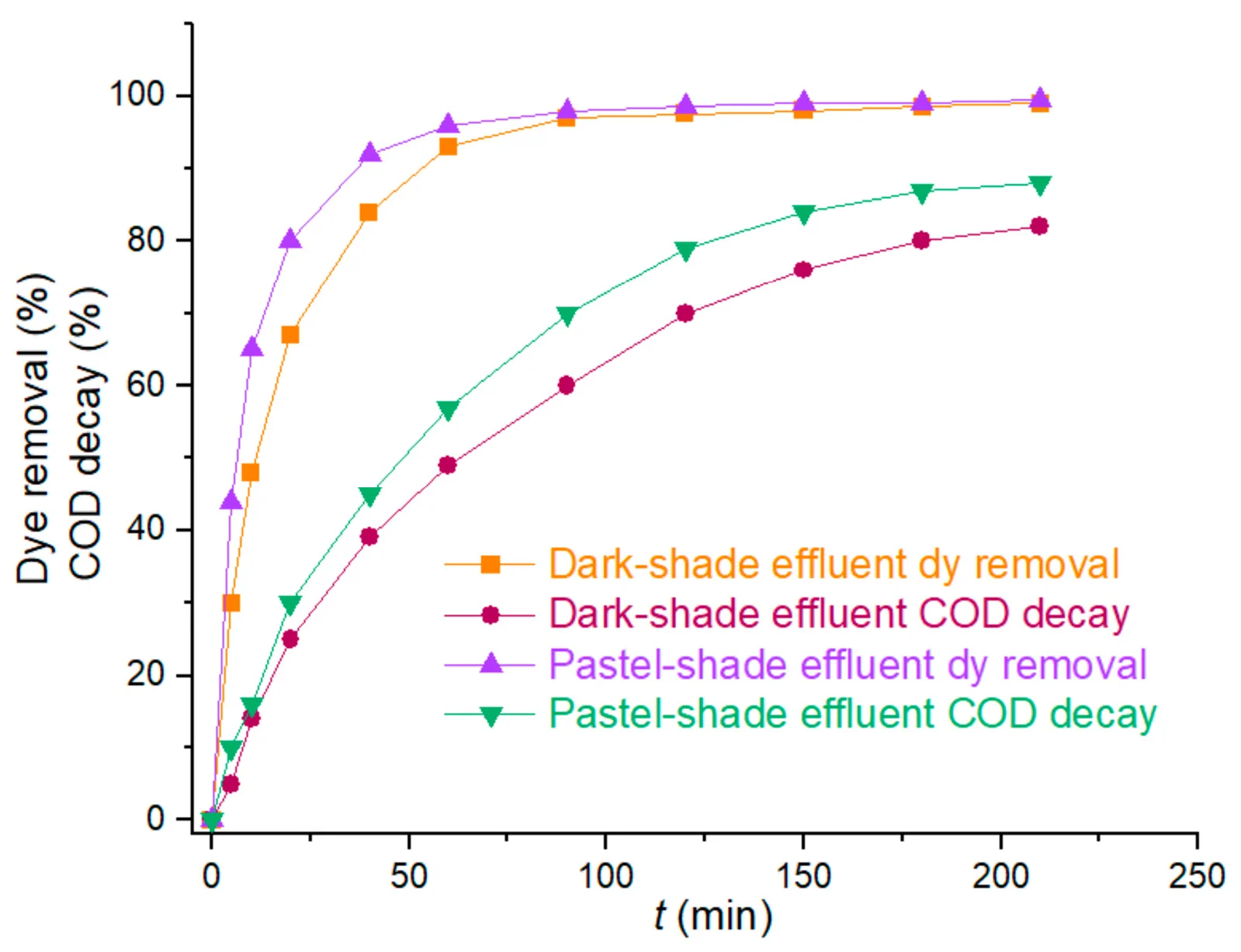
Degradation of two textile dyeing effluents containing RB 4 dye (40 mA cm^−2^, all experiments performed in triplicate; estimated error within ±2%).

Complete decolorization was achieved in about 70 and 110 min, and COD decay was about 87% and 80% after 180 min of electrolysis for pastel- and dark-shade effluents, respectively. These were approximately the equilibrium values for both effluents, indicating high organic matter removal, i.e., high mineralization. Further COD decay changed very little over another 60 min of electrolysis (Figure 7). Energy consumption was 1.75 kWh m^−3^ and 2.39 kWh m^−3^ for complete decolorization and 2.97 kWh m^−3^ and 2.91 kWh m^−3^ for the measured COD decay after 180 min of electrolysis of pastel- and dark-shade effluents, respectively. The corresponding pH and electrical conductivity after finishing the electrolysis were 11.21 and 11.02 and 191 mS cm^−1^ and 196 mS cm^−1^ for pastel- and dark-shade effluents, respectively. They remained relatively constant, which was expected, taking into account the relatively high concentrations of sulphate and carbonate.

Oxidative dye degradation at the graphite–Co_3_O_4_ anode was performed in real textile effluent, similar to many reported in the literature and in practical guides [3,27,28]. It was previously shown that the initial concentration of the dye and salt (Na_2_SO_4_ in this case), as well as the pH, affected the degradation rate and efficiency. The decolorization rate decreased with the initial dye concentration; therefore, the decolorization of the pastel-shade effluent was faster than the decolorization of the dark-shade one. Both pastel- and dark-shade effluents were completely decolorized, so electrolysis with the graphite–Co_3_O_4_ anode exhibited high efficiency.

The initial dye concentration and pH of the pastel-shade effluent were similar to those of a model dye solution used for examining the effect of alkaline pH on decolorization (53 mg dm^−3^ and 50 mg dm^−3^ of RB 4 and pH 11.54 and 11.0, respectively). The Na_2_SO_4_ concentration in both samples was in the range where it had almost no effect on degradation (about 0.7 mol dm^−3^ and 0.5 mol dm^−3^ for the pastel-shade effluent and the model solution mentioned, respectively; Figure 6c). Thus, the rate and efficiency of the electrochemical degradation of these two samples using the graphite–Co_3_O_4_ anode were analyzed and compared. As the model dye solution was decolorized in about 40 min, it took approximately another 30 min to decolorize the effluent. The difference in the initial dye concentration between the two samples was small enough to be neglected. The difference in pH was relatively small but not negligible; it could affect the decolorization rate and thus be the reason for the slower decolorization of the effluent. A significant difference between the two samples is the presence of carbonates in the effluent. The HCO_3_^−^ ions, generated by their dissociation in water, reacted with the hydroxyl radicals, forming the HCO_3_· radicals, which were much weaker than ·OH radicals [43]:

CO_3_^2−^ + H_2_O → HCO_3_^−^ + OH^−^ (16)

HCO_3_^−^ + ·OH → HCO_3_ + OH^−^ (17)

This way, HCO_3_^−^, i.e., CO_3_^2−^, acted as the·OH radical quencher, and it was another reason for the reduced degradation rate of effluent compared to the similar model solution. Despite that, the RB 4 was efficiently removed from the real dyeing effluent using the graphite–Co_3_O_4_ anode with relatively low energy consumption (Table 2).

##### Regeneration and Reuse of the Anode

**Figure 8:**
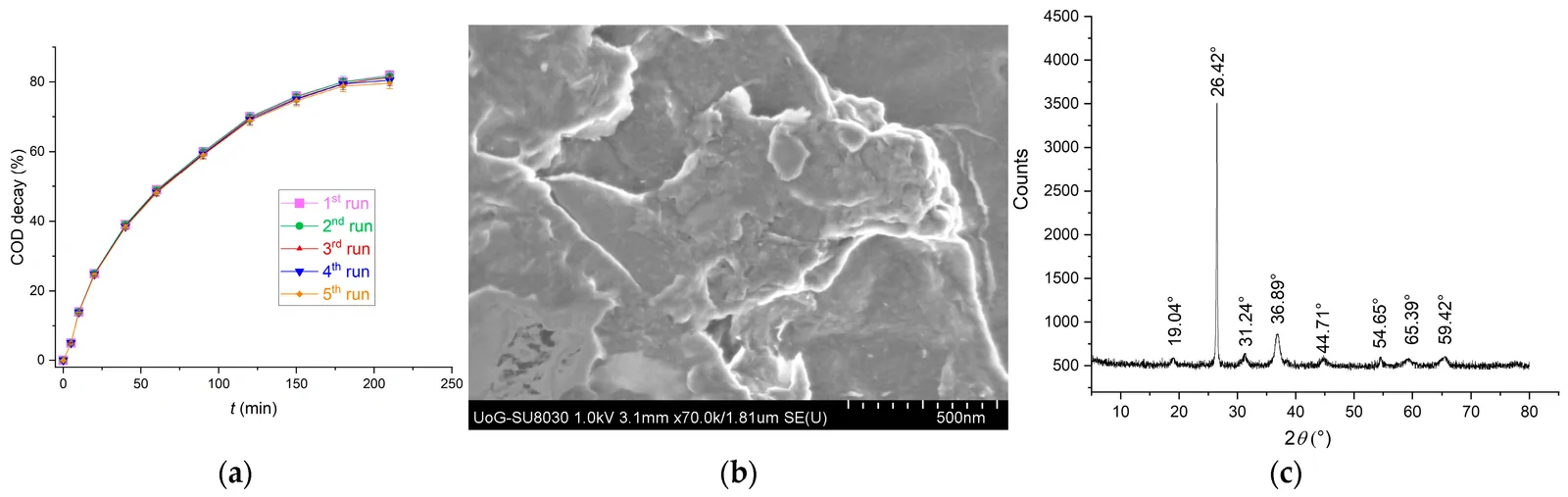
(**a**) COD decay, (**b**) SEM image, and (**c**) XRD pattern of the graphite–Co_3_O_4_ anode after 5th consecutive electrolysis session of the dark-shade textile effluent (40 mA cm^−2^; all experiments performed in triplicate; estimated error within ± 2%).

Degradation of the textile effluent was repeated five consecutive times to check the stability of the anode. After each experiment, the anode was regenerated by washing with distilled water and ethanol and drying in air at 80 °C. Each experiment was repeated, and the loss of the anode’s activity was very low (it maintained about 97.5% of its initial activity for COD decay after five consecutive runs of the treatment of the dark-shade effluent (Figure 8a); this also supports the presumption of the electron exchange between the dye and cobalt ions, which likely regenerate at the anode surface during the electrolysis). Although it could be said that the differences are mostly within the measurement error, the trend of very slight activity loss can be observed. Cobalt was not detected in either solution after electrolysis experiments (atomic absorption spectroscopy (AAS), detection limit about 5 µg dm^−3^).

The anode’s surface morphology does not seem to be notably changed after the 5th experimental run (SEM image, Figure 8b). EDX analysis showed no significant difference in the elemental composition and distribution (11 to 16% Co and 17 to 33% O). Traces of S, which probably originated from the sulphate in the effluent, were detected on the anode’s surface. They obviously did not notably affect the anode’s activity in the later runs. The anode also maintained its crystal structure (XRD pattern Figure 8c), so, overall, it remained stable during the experiments, i.e., no significant changes were detected by the characterization techniques used.

## 3. Materials and Methods

### 3.1. Materials

Discarded graphite tubes for AAS samples (graphite tubes in the following text) served as the starting material for both non-modified and modified electrodes. CoSO_4_·7H_2_O, RB 4 (Table 4), H_2_SO_4_, Na_2_SO_4_, NaOH, Na_2_CO_3_ (soda ash), and ethanol were purchased from Sigma-Aldrich (St. Louis, USA) and used without further purification.

### 3.2. Preparation and Characterization of the Electrode

Two graphite tubes (total active geometrical surface 10.2 cm^2^ each) were ultrasonically rinsed with 1 M H_2_SO_4_, deionized water, and methanol and then dried in air at room temperature. One of them was electrochemically modified. All electrochemical experiments (electrodeposition and electrooxidation) were carried out using an Amel 510 potentiostat (Materials Mates, Milano, Italy), controlled by VoltaScope 5.0 software. Electrodeposition was performed in a conventional two-electrode, one-compartment cell containing 0.05 M CoSO_4_·7H_2_O and 0.001 M H_2_SO_4_, with the graphite tube serving as the cathode and Pt as the anode at a current density of 30 mA cm^−2^ for 10 min (the current density and the electrodeposition time were the key factors affecting the Co_3_O_4_ loading on the graphite surface, and they were established after the previous research, which showed that these values provided the anode with the highest exhibited activity). The modified graphite tube was then washed with deionized water, air-dried at 200 °C for 60 min, and used as the anode in electrooxidation experiments.

Secondary electron emission (SEM) images were collected using a Hitachi High Technologies SU8030 (Tokyo, Japan). The field emission gun operated at an accelerating voltage of 1 kV. Samples were attached to aluminum stubs with Leit adhesive carbon tabs. EDX analysis was conducted on a Zeiss Sigma 300 (Cambridge, UK) at 20 kV with an Oxford Instruments EDX detector (Abingdon, UK). XRD was performed on a Bruker D8 Advance X-ray Diffractometer (Bruker-AXS, Karlsruhe, Germany) in theta–theta geometry in reflection mode. Cu Kα radiation was used at 40 kV and 40 mA with a LynxEye (Karlsruhe, Germany) silicon strip position-sensitive detector. Data were collected between 2° and 80° 2*θ*, with a step size of 0.02°, and a counting time of 0.5 s per step, using DIFFRAC plus XRD Commander version 2.6.1 software (Bruker-AXS), qualitative assessment with the aid of EVA version 5 (Bruker-AXS), and the PDF-2 2024 database (ICDD). The pH_pzc_ was determined by potentiometric titration. FTIR spectra were recorded using a BOMEM MB-100 FTIR spectrometer (Hartmann & Braun, Quebec, QC, Canada), using KBr pellets containing 1.0 mg of the sample (material peeled from both electrodes’ surfaces, dried at 100 °C, and ground)/150 mg KBr. The instrument used a standard DTGS/KBr detector (4000–400 cm^−1^, resolution 2 cm^−1^). For the BET analysis, the electrode surface material samples were peeled off, ground, and degassed overnight at 40 ◦C under flowing N_2_. BET data were collected on a Micromeritics Gemini VI Surface Area Analyzer (Norcross, GA, USA). Electrochemical characterization was performed using the mStat4S potentiostat (PalmSens, Houten, The Netherlands), with Ag/AgCl and Pt as the reference and auxiliary electrodes, respectively. Graphite and graphite–Co_3_O_4_ anodes were ultrasonically cleaned in ethanol, washed with deionized water, and electrochemically cleaned by cyclic voltammetry in 0.1 mol/dm^3^ H_2_SO_4_ over the potential range from −1.0 to +1.0 V at a scan rate of 100 mV/s. The electrochemically active surface area (ESA) of the electrodes was determined by cyclic voltammetry in an equimolar mixture of hexacyanoferrate(II) and hexacyanoferrate(III) with a total concentration of 10 mmol/dm^3^, at scan rates of 10, 15, 20, 25, and 30 mV/s. The ESA was calculated using the Randles–Ševčík equation:

(18) $$i_{p}={2.69\cdot 10}^{5}\cdot n^{3/2}\cdot A\cdot c_{0}\cdot D^{1/2}\cdot v^{1/2}$$

where *i*_p_ is the peak current (A), *n* is the number of transferred electrons (1 in our case), *A* is the electroactive surface area (cm^2^), *c*_0_ is the electroactive species concentration, *D* is the diffusion coefficient (7.6 × 10^−6^ cm^2^/s), and *υ* is the scan rate (V/s).

The pH_pzc_ of the graphite–Co_3_O_4_ anode’s surface was determined by potentiostatic titration.

### 3.3. Electrochemical Oxidation Experiments

To examine the electrochemical oxidation process mechanism, kinetics, and parameters, electrolysis was performed on model dye solutions prepared by diluting the stock solution to the required concentration (stock solution was prepared by dissolving 1000.0 mg RB 4 in 1000 cm^3^ of deionized water) and then dissolving the required amount of Na_2_SO_4_. Electrooxidation was performed at a constant current density of 40 mA cm^−2^ (except for the current density experiments) and a native pH of 6.8 (except for the pH experiments). Modified and non-modified graphite tubes were attached as the anodes in separate experiments (with a Pt cathode).

For dye electrooxidation under real conditions, the dyebath was prepared according to the references [27,28]. It contained RB 4 (500 mg dm^−3^ and 1500 mg dm^−3^ for pastel and dark shades, respectively), Na_2_CO_3_ (50 g dm^−3^), Na_2_SO_4_ (100 g dm^−3^), and cotton fabric for dyeing, previously washed and bleached (100 g dm^−3^). Dyeing was performed at 40 °C for 30 min (for pastel shade) and 45 min (for dark shade). The dyed fabric was then removed, and the electrolysis of the remaining dyebath was performed at a constant current density of 40 mA cm^−2^.

The dye concentration was determined at 598 nm (λ_max_) using a UV-1650 PC UV-vis spectrophotometer (Shimadzu, Kyoto, Japan). The pH was adjusted by adding H_2_SO_4_ and NaOH and measured by the SensIon3 pH meter (Hach, Loveland, CO, USA). Chemical oxygen demand (COD) was determined using the kit consisting of the thermoreactor RD125, the Lovibond^®^ COD Vario tube test, and the Lovibond^®^ Multidirect photometer (Lovibond, Sarasota, FL, USA). The cobalt concentration in the solutions was measured using atomic absorption spectroscopy (AAS, Analyst 300, PerkinElmer, Shelton, CT, USA).

The dye removal efficiency was presented as the percentage (%) of the dye removed at time *t*.

The COD decrease was expressed as a percentage (%) of the initial COD removed at the treatment time *t*.

Energy consumption *E_c_* (kWh m^−3^) was calculated from Equation (19):

(19) $$E_{c\;=\;\frac{UIt}{1000V}}$$

where *I*, *t*, *U*, and *V* are the current (A), time (h), average voltage (V), and volume of wastewater (m^3^) [2,9].

## 4. Conclusions

Modification of the graphite surface with Co_3_O_4_ improved its performance as the anode for electrochemical oxidation of the organic compound. The decolorization of the RB 4 dye solution proceeded more than 5 times faster, the corresponding energy consumption was 3.67 times lower, and the equilibrium COD decay was 55% higher for the graphite anode modified by Co_3_O_4_ than for the non-modified one. The principal degrading agent generated at both modified and non-modified graphite surfaces was an ·OH radical. There is an indication (based on the indirect evidence obtained by several different independent experiments) that the Co^3+^/Co^2+^ redox couple was also significantly involved in the electrochemical processes at the modified anode via electron exchange, facilitating dye degradation. A Co_3_O_4_–modified anode was successfully tested for the treatment of the textile dyeing effluent with two different dye concentrations. Both effluents were completely decolorized, reaching an equilibrium COD decay of over 80%, with very acceptable energy consumption (compared to the literature results). The anode was efficient in a relatively wide pH range, from acidic to alkaline, and remained stable in the electrolysis conditions. The results are very encouraging regarding the modifications of the simple, well-known, and recyclable materials to obtain new ones, with improved features for a particular purpose. The prospects of this work would be further and deeper research of the interactions at the described surfaces and the application of the results to similar systems to improve a specific surface reaction.

## Figures and Tables

**Table 1:** BET surface area, pore volume, and pore size of the graphite and graphite–Co_3_O_4_ anodes.

Anode	BET Surface Area (m^2^ g^−1^)	Pore Volume		Pore Size	
		BJH Adsorption Cumulative Volume of Pores Between 17.000 Å and 3000.000 Å Diameter (cm^3^ g^−1^)	BJH Desorption Cumulative Volume of Pores Between 17.000 Å and 3000.000 Å Diameter (cm^3^ g^−1^)	BJH Adsorption Average Pore Diameter (4 V/A) (Å)	BJH Desorption Average Pore Diameter (4 V/A) (Å)
Graphite	3.6461	0.010688	0.010539	108.444	86.800
Graphite–Co_3_O_4_	11.4438	0.033121	0.033105	110.090	92.816

**Table 2:** The performance of the graphite–Co_3_O_4_ anode and other relevant anodes in similar electrochemical dye degradation processes.

Anode Type	Compound, Experimental Conditions	Achievement	Energy Consumption (kWh m^−3^)	Reference Number
MMO-coated Ti	Direct Blue 86 (50 mg dm^−3^), pH = 5; CD = 15 mA cm^−2^	98.03% dye removal; 95% COD removal in 105 min	5.61	[2]
BDD	Rhodamine B (50 mg dm^−3^) pH = 7, *j* = 40 mA∙cm^−2^, *c* (Na_2_SO_4_) = 0.035 mol·dm^−3^	Removal efficiency 93.83% in 60 min	32.03	[5]
Pt BDD	Methyl Violet (100 cm^3^, 100 mg dm^−3^ TOC), 0.05 M Na_2_SO_4_ at 35.0 °C, pH 3, 33.3 mA cm^−2^	49% color removal in 600 min (92% at 100 mA∙cm^−2^, Pt); 100% color removal in 150 min; 74% mineralization in 360 min (BDD)	* 0.60 (kW h (g TOC)^−1^) for 74% demineralization on BDD anode	[8]
Graphite	Acid Black 210 (15 mg dm^−3^), 2.5 g dm^−3^ NaCl 20 mA cm^−2^, pH 6.63	95% degradation for 609 nm in 10 min	4.61	[9]
Ti-PbO_2_	Allura Red AC Erythrosine B (100 mg dm^−3^ each), 0.05 M Na_2_SO_4_, I = 1 A (prm 800, US freq = 40 kHz for sonoelectrooxidation)	100% color removal in 90 min (electrooxidation, Ech); in 60 min (sonoelectrooxidation, Sech)	For 50% COD decrease: Allura Red: about 5 (Sech) and 8 (Ech); Erythrosine B: about 8 (Sech) and 12.5 (Ech)	[12]
IrO_2_/Ti	Basic Yellow 28 (2 × 10^−5^ mol dm^−3^), 30 mA cm^−2^, pH 2.5, 0.08 M Na_2_SO_4_/pH 3, 0.03 M NaCl	92.9% decolorization, 26.8% COD removal for Na_2_SO_4_; 93.8% decolorization, 46.2% COD removal for NaCl in 15 min	* 76.9 kWh (g COD)^−1^	[14]
Pt/CuNPs	Congo Red (50 mg dm^−3^), 0.25 M KCl, pH 7.5, 2V vs. Ag/AgCl	Degradation 95.95% in 60 min	About 70	[17]
Cu-MOF/Pt	Tartrazine dye (50 mg dm^−3^), 2 V, pH 3, 02M KCl	99.8% decolorization in 20 min	0.0834	[18]
Porous-Ti/BDD	X-GN dye (100 mg dm^−3^), 50 mA cm^−2^, 100 mM Na_2_SO_4_, pH not adjusted, t = 60 °C	Complete decolorization, 69.24%; TOC removal in 50 min	5.62	[37]
Graphite–Co_3_O_4_	Reactive Blue 4 (50 mg dm^−3^), *C*_0_ (RB 4) = 50 mg dm^−3^, *j* = 40 mA cm^−2^, *c* (Na_2_SO_4_) = 0.5 mol dm^−3^, pH = 6.8	100% color removal in 40 min; 91% COD removal in 180 min	0.89 3.19	This work

* The unit is (g COD)^−1^ just in this case (instead of kWh m^−3^ as for the rest of the data).

**Table 3:** Kinetic parameters of the RB 4 decolorization on the graphite–Co_3_O_4_ anode.

*C*_0_ (mg dm^−3^)	*k*_1_ (min^−1^)	*R* ^2^
20	0.0813	0.9859
50	0.0753	0.9839
100	0.0642	0.9798
200	0.0505	0.9776

**Table 4:** RB 4 dye properties.

RB 4 Molecular Structure	Synonym	Molecular Weight (g mol^−1^)	λ_max_ (nm)
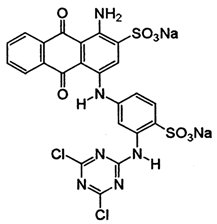	Procion^®^ blue MX-R	637.4	598

## Data Availability

The original contributions presented in this study are included in the article and Supplementary Materials. Further inquiries can be directed to the corresponding author.

## Supplementary Materials

The following supporting information can be downloaded at: https://www.mdpi.com/article/10.3390/molecules31162785/s1, Figure S1a: EDX spectrum of the Graphite–Co_3_O_4_ anode (representative surface); Figure S1b: Mapping of the elements on the examined Graphite–Co_3_O_4_ anode surface; Figure S2: Radical scavenger test results of the Graphite and Graphite–Co_3_O_4_ anodes for the degradation of RB 4 dye (*C*_0_ (RB 4) = 50 mg dm^−3^, *j* = 40 mA cm^−2^, *c* (Na_2_SO_4_) = 0.5 mol dm^−3^, and pH = 6.8; all experiments were performed in triplicate; estimated error within ±2%); Figure S3: Dye degradation under an Ar-saturated atmosphere at the Graphite and Graphite–Co_3_O_4_ anodes for the degradation of RB 4 dye (*C*_0_ (RB 4) = 50 mg dm^−3^, *j* = 40 mA cm^−2^, *c* (Na_2_SO_4_) = 0.5 mol dm^−3^, and pH = 6.8; all experiments were performed in triplicate; estimated error within ±2%).
