# Supplementary material for: Modifying Recycled Graphite with Co3O4 Towards a Novel, Efficient Anode for the Electrochemical Treatment of a Textile Dyebath
Source: Molecules. 2026 Aug 10;31(16):2785. doi: 10.3390/molecules31162785 (PMC13515682; doi:10.3390/molecules31162785)
Supplement: Supplementary file 1 [file molecules-31-02785-s001.zip › molecules-4427242-supplementary.pdf]

Supplementary Figure Captions

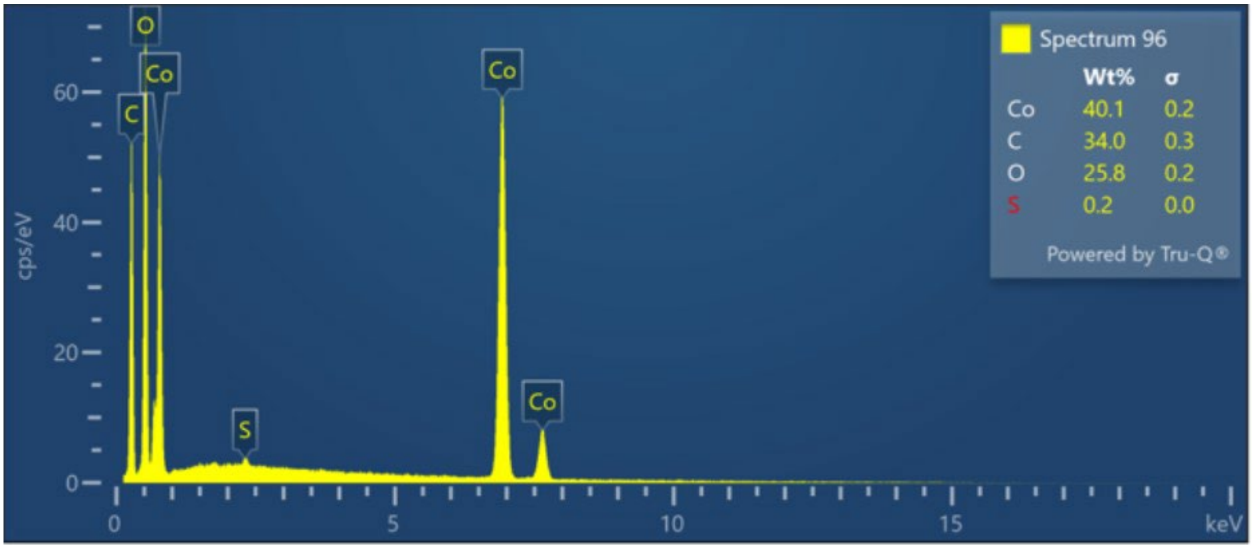

Figure S1a. EDX spectrum of the graphite–Co_3O_4 anode (representative surface).

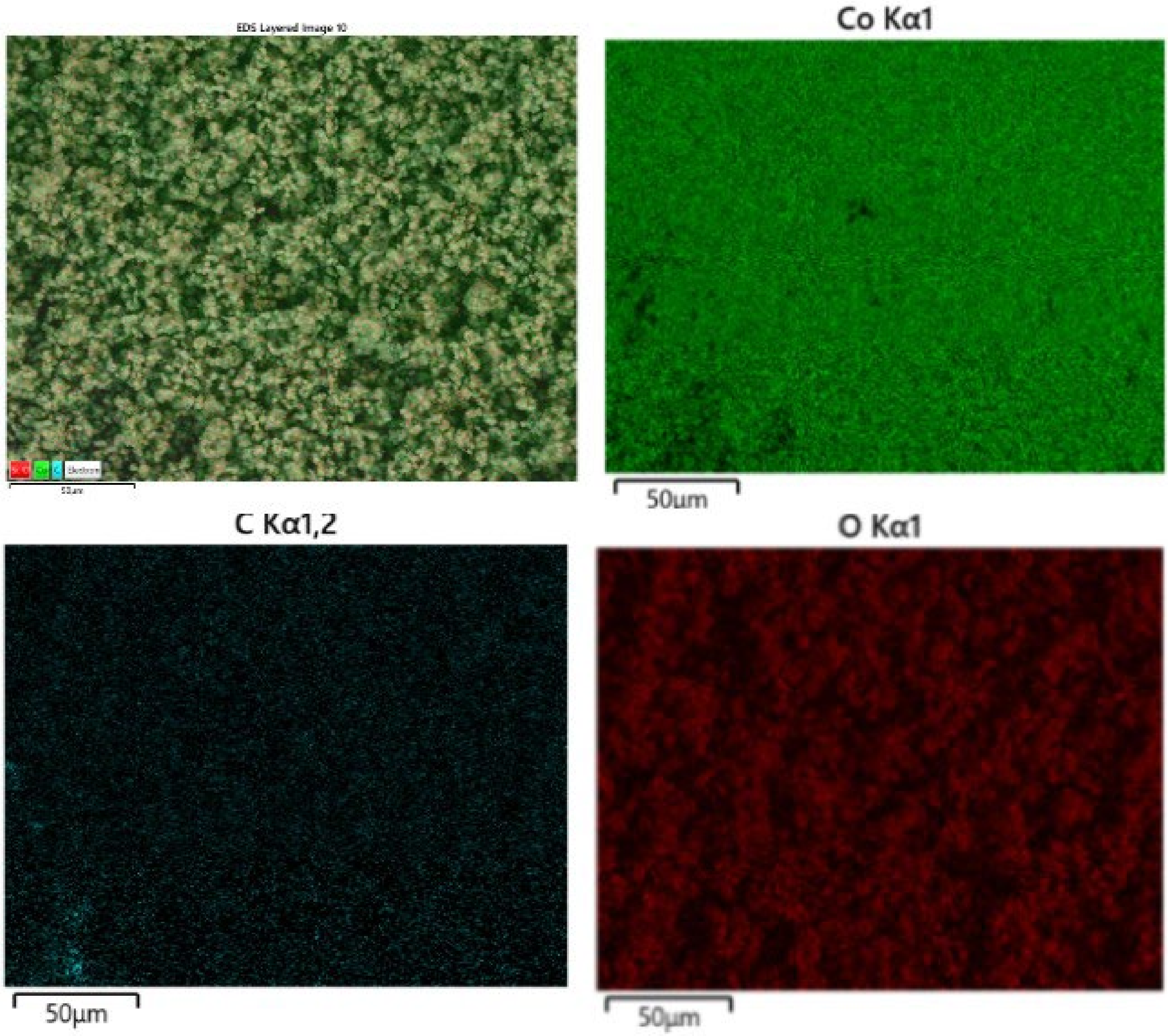

Figure S1b. Mapping of the elements on the examined graphite–Co3O4 anode surface.

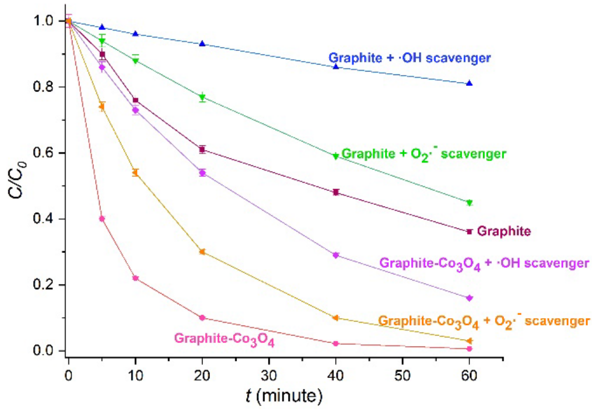

Figure S2. Radical scavenger test results of the graphite and graphite–Co_3O_4 anodes for the degradation of RB 4 dye (C_0 (RB 4) = 50 mg dm^{-3}, j = 40 mA cm^{-2}, C (Na_2SO_4) = 0.5 mol dm^{-3}, and pH = 6.8; all experiments were performed in triplicate; estimated error within ±2%).

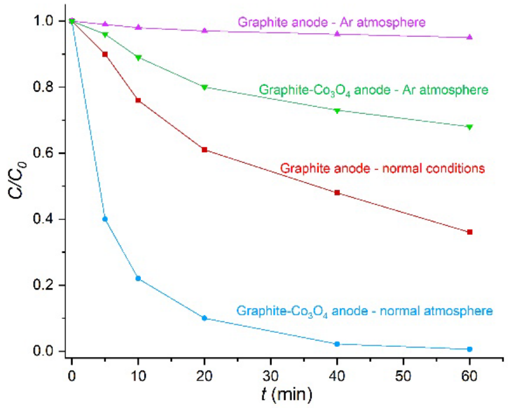

Figure S3. Dye degradation under an Ar-saturated atmosphere at the graphite and graphite–Co_3O_4 anodes for the degradation of RB 4 dye (C_0 (RB 4) = 50 mg dm^{-3}, j = 40 mA cm^{-2}, C (Na_2SO_4) = 0.5 mol dm^{-3}, and pH = 6.8; all experiments were performed in triplicate; estimated error within ±2%).
